# Medical Items

**Published:** 1911-05

**Authors:** 


### MEDICAL ITEMS

### THE IMPORTANCE OF TONIC MEDICATION.

The more one studies disease and the phenomena which attend what some one has called the "unphysiological state," the more evident it becomes that the basis of the great majority of human affections is a decrease or perversion of bodily functions. Inasmuch as this is so apparent from even the most superficial investigation of the disorders of digestion, nutrition and elimination, the wonder is that more attention has not been given to increasing functional activity and restoring the physiological balance. Unquestionably the keynote of any successful treatment of depraved or perverted function of tissue cells or the special organs, is appropriate stimulation, using ways and means that clinical experience has shown capable of producing effects that are not only prompt and positive but also prolonged and substantial.

